# Mediastinal pancreatic pseudocyst with hemorrhage and left gastric artery pseudoaneurysm, managed with left gastric artery embolization and placement of percutaneous trans-hepatic pseudocyst drainage

**DOI:** 10.1093/gastro/gou084

**Published:** 2014-12-09

**Authors:** Parag Brahmbhatt, Jason McKinney, John Litchfield, Mehul Panchal, Thomas Borthwick, Mark Young, Lance Klosterman

**Affiliations:** ^1^Division of Gastroenterology and Hepatology, East Tennessee State University, Johnson City, TN, USA,; ^2^Department of Medicine, M. P. Shah Medical College, Jamnagar, Gujarat, India,; ^3^Department of Gastroenterology, James H. Quillen VA Medical Center, Johnson City, TN, USA and; ^4^Department of Radiology, James H. Quillen VA Medical center, Johnson City, TN, USA

**Keywords:** mediastinal pancreatic pseudocyst, dysphagia, percutaneous pseudocyst drainage

## Abstract

Mediastinal pancreatic pseudocyst (MPP) is a rare, but known, complication of both acute and chronic pancreatitis. Most pseudocysts are associated with alcoholic pancreatitis. Recent advances in endoscopic techniques have shown promising results, with reduced chances of infection and recurrence than with percutaneous drainage, but limited availability restricts widespread use. Left gastric artery pseudoaneurysm with mediastinal pseudocyst has not been described in the literature to date. We report a successful resolution of hemorrhagic MPP with embolization of pseudoaneurysm and percutaneous trans-hepatic pseudocyst drainage.

## Introduction

The formation of a pseudocyst is a common complication of both acute and chronic pancreatitis, in which fluid is encased by a wall of inflamed tissue. Pseudocysts most commonly occur in the abdomen; however, extension into the mediastinum does occur infrequently and can be a rare cause of dysphagia, secondary to extrinsic compression of the esophagus. We report a case presented with dysphagia and epigastric discomfort.

## Case presentation

A 60-year-old male patient, with a history of alcohol abuse, was admitted with acute pancreatitis attributed to alcohol. This was his first admission for pancreatitis. Computed tomography (CT) demonstrated acute fluid collections extending along the long axis of the pancreas. The patient improved with conservative treatment measures and was discharged home. Outpatient appointment was planned but the patient failed to follow up. He was advised to abstain from alcohol but continued daily drinking.

He returned three months after discharge following three days of dysphagia to both solids and liquids. The patient also mentioned that swallowing produced a localized, intense pain in his epigastric area, which was not similar to his previous episode of pancreatitis. The patient denied fever, chills, nausea or vomiting.

On physical examination, the patient was afebrile and his vital signs were within normal limits. He appeared cachectic. Neither jaundice nor scleral icterus were noted. His abdomen was neither distended nor tender to palpation. Complete blood count and comprehensive metabolic panel were within normal limits, with the exception of hemoglobin of 9.5 g/dL, which was unchanged from previous levels. Amylase and lipase were 483 and 2125 U/L, respectively.

**Figure 1.**
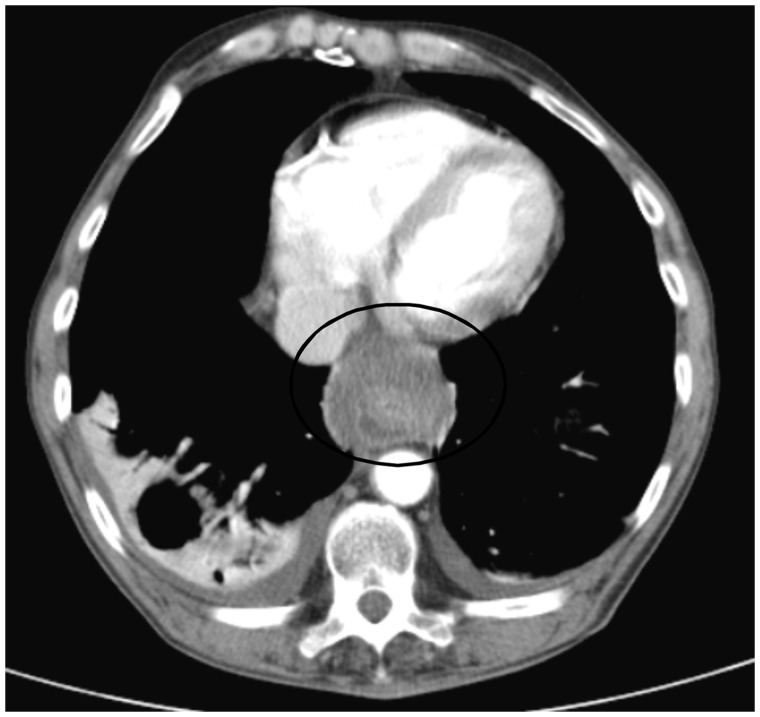
Circumferential lower periesophageal fluid collection from the superior extension of the pancreatic pseudocyst containing hemorrhage.

**Figure 2.**
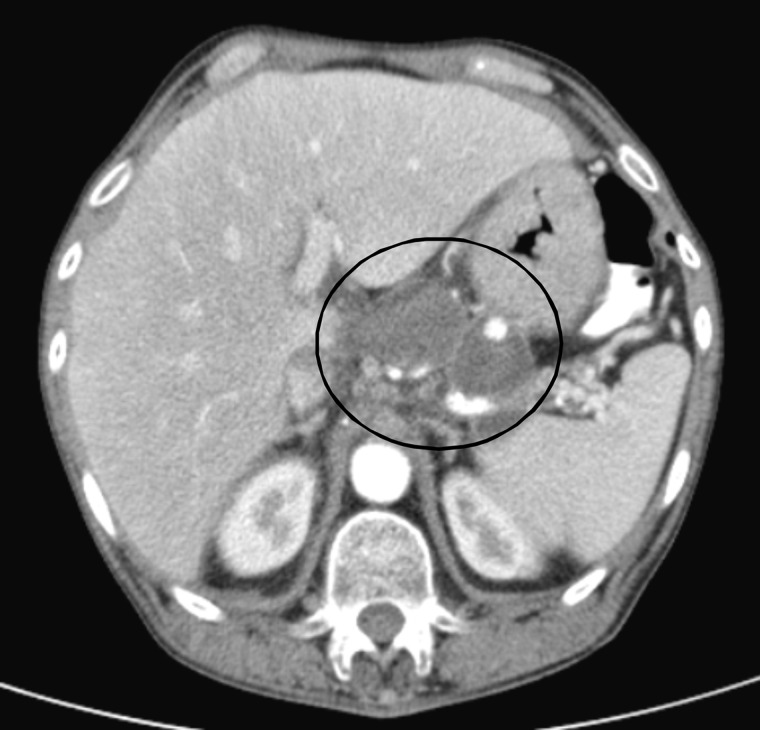
Intrapancreatic/peripancreatic pseudocysts along the pancreatic tail and body demonstrating a new central density of 33 Hounsfield units compatible with hemorrhage in the pseudocyst.

**Figure 3.**
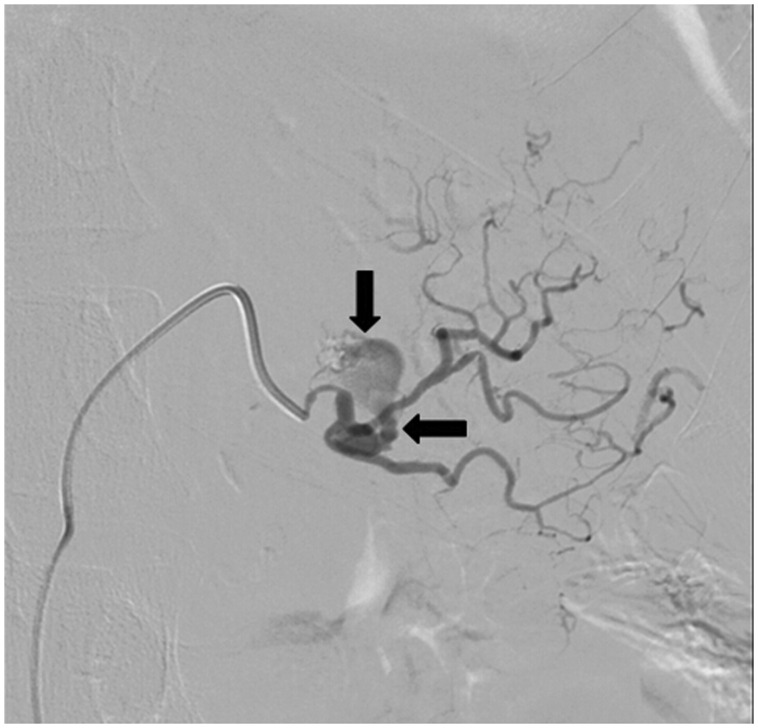
Pseudoaneurysm originating from the branch of left gastric artery

CT scan with contrast showed a circumferential lower periesophageal fluid collection (Figure 1) which demonstrated hazy increased density compatible with the mediastinal extension of a pancreatic pseudocyst containing hemorrhagic fluid. It also showed a new, central enhancing density within previously present intrapancreatic/peripancreatic pseudocysts (Figure 2) compatible with casts of blood, along with rapidly increasing left gastric artery pseudoaneurysm measuring 9 mm across, compared with 3 mm on CT scan performed 2 months previously (Figure 3). Small bilateral dependent pleural effusions were also present.

**Figure 4.**
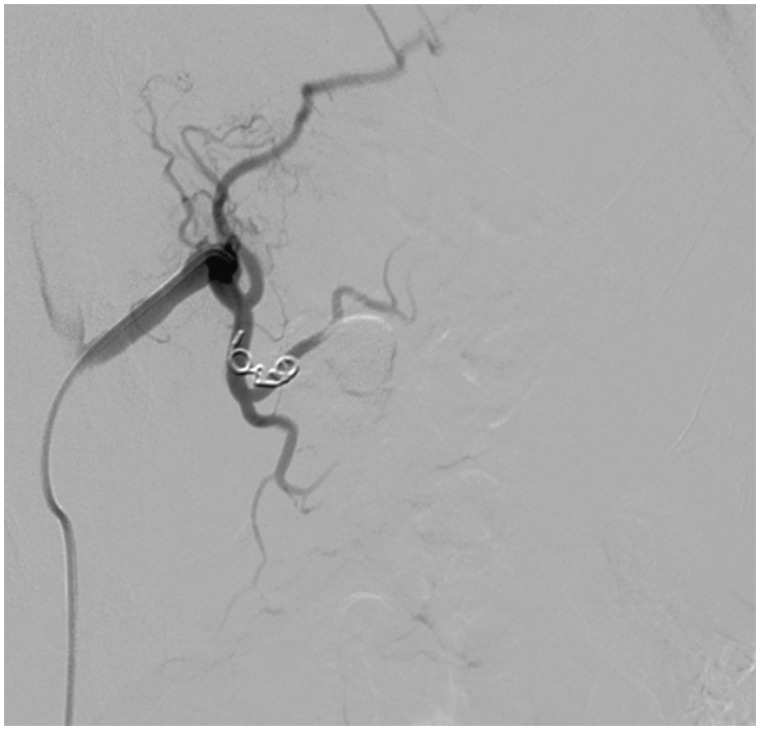
Successful embolization of the pseudoaneurysm with Gelfoam pledgets and Tornado coils.

**Figure 5.**
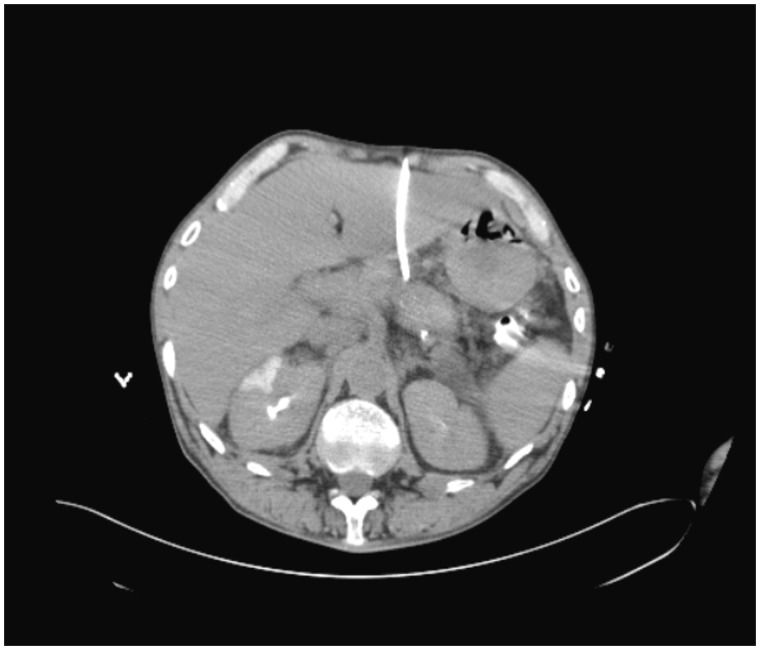
Percutaneous trans-hepatic pseudocyst drainage.

The decision was made to drain the pseudocyst, but the left gastric artery pseudoaneurysm first had to be embolized in order to prevent hemorrhagic complications. The patient was taken to the catheterization laboratory and an angiogram was performed, which revealed a large pseudoaneurysm supplied by a branch of the left gastric artery. This branch was selected with the 4-French catheter. Three separate Gelfoam pledgets were deployed and two separate Tornado coils were placed. Follow-up angiogram demonstrated successful embolization of the pseudoaneurysm (Figure 4). The patient then underwent CT-guided percutaneous trans-hepatic drainage of the pseudocyst with placement of 10-French draining catheter (Figure 5). The patient’s dysphagia dramatically improved and repeat CT scan showed resolution of the mediastinal pseudocyst.

## Discussion

There have been some recent changes in the classification and management of pancreatitis and its complications. The Atlanta Classification of acute pancreatitis, which was originally described in 1992, was revised in 2012. According to the revised Atlanta Classification, pancreatic pseudocyst is defined as an encapsulated collection of fluid with a well-defined inflammatory wall usually outside the pancreas with minimal or no necrosis [1]. Pancreatic pseudocyst usually develops 4 weeks after the onset of acute pancreatitis. The fluid collection up to the 4-week point is usually described as ‘peripancreatic fluid collection' and is defined as peripancreatic fluid associated with interstitial edematous pancreatitis without associated peripancreatic necrosis [1].

While reported incidence of pancreatic pseudocyst varies in the literature, it is described as 7–25% with acute pancreatitis and 20–25% with chronic pancreatitis [2]. Pancreatic pseudocyst is further classified into intrapancreatic and extrapancreatic, based on anatomical location [3]. Intrapancreatic pseudocysts are usually small in size. Extension of the pseudocyst into the adjacent viscera—including spleen, liver, transverse colon, anterior or posterior pararenal space, retroperitoneaum, and mediastinum—has been described in the literature, which contains about 50 reported cases of mediastinal extension of pseudocyst [2, 4].

The pathogenesis of pseudocyst formation in acute and chronic pancreatitis is well recognized. Acute pseudocyst formation occurs as a result of the accumulation of pancreatic secretions along with the products of the inflammatory response, with or without pancreatic duct damage [3]. As described earlier, an acute fluid collection extending along the peripancreatic and pararenal boundaries is known as a peripancreatic fluid collection and, when this persists for more than 4 weeks, along with formation of a well-defined wall of fibrous or granulation tissue, a pancreatic pseudocyst forms [1, 3]. Pancreatic duct disruption and pancreatic duct obstruction secondary to calculus, protein plug or localized stricture also play a major role in the formation of chronic pseudocyst [3, 4].

Mediastinal extension is thought to be caused by rupture of the pancreatic duct posteriorly into the retroperitoneal space. The fluid then travels through the esophageal and aortic openings in the diaphragm to gain access to the posterior mediastinum [4–6]. The most common presenting feature of mediastinal pancreatic pseudocyst (MPP) is dysphagia secondary to compression of esophagus. Most patients also complain of dyspnea secondary to accompanying pleural effusion [6–9]. Our patient also had bilateral pleural effusions. Most patients also experience weight loss secondary to dysphagia. Other clinical manifestations include abdominal pain, chest pain, palpitation, odynophagia and, rarely, it can also mimic pseudoachylasia [4, 10].

While chest radiography is the initial test in the majority of the patients presenting with dyspnea and dysphagia, it is not very useful in the diagnosis of mediastinal pancreatic pseudocyst: t he majority of these are diagnosed by CT scan. Contrast-enhanced CT scan has a very high sensitivity and it also helps to evaluate the pancreas the and anatomical relationship of pancreatic pseudocyst to the surrounding structures. Although magnetic resonance imaging (MRI) and magnetic resonance cholangiopancreatography (MRCP) will provide further details of pancreatic ductal anatomy, CT scan by itself is diagnostic in the majority of cases [2, 4]. Recent advances in endoscopic techniques, such as endoscopic ultrasound (EUS), are helpful not only in evaluating the mediastinal mass and cysts, but also play an important therapeutic role by allowing EUS-guided aspiration and drainage of the cyst [6, 11].

As with other diseases, MPP has its own set of complications, including infection, hemorrhage into the cyst, or manifestations secondary to compression and invasion of nearby anatomical structures. Life-threatening complications, such as cardiac tamponade, hypotensive shock and respiratory failure, have also been described in the literature [12–14].

Management of MPP depends on their size, number, location, relationship with surrounding anatomical structures, severity of symptoms, presence or absence of infection, and presence of communication of the cyst with the pancreatic duct [2, 15]. Spontaneous resolution has been described in the literature but is very rare [16, 17]. Successful resolution of the cyst after medical management, such as use of total parenteral nutrition, octreotide and bromhexine hydrochloride, has also been reported (Table 1) [16–25].

**Table 1.** Reported cases of successful resolution after medical management

No.	References	Age (years); gender	Management
1	Leechawengwong *et al.* 1979 [16]	39; male	Observation
2	Frenzer *et al.* 1995 [18]	59; male	Total parenteral nutrition
3	Singh *et al.* 1996 [19]	40; male	Octreotide
4	Ishibashi *et al.* 1999 [20]	(N/A)	Somatostatin analog
5	Yasuda H *et al.*, 1999 [21]	43; male	Octreotide
6	Akashi *et al.* 2003 [22]	(N/A)	Somatostatin analog
7	Tsujimoto *et al.* 2004 [23]	49; male	Bromhexine hydrochloride
8	Sakamoto *et al.* 2007 [24]	51; male	Total parenteral nutrition
9	Santoshkumar *et al.* 2007 [17]	39; male	Alcohol abstinence
10	Panackel *et al.* 2008 [25]	35; male	Octreotide and total parenteral nutrition

**Figure 6.**
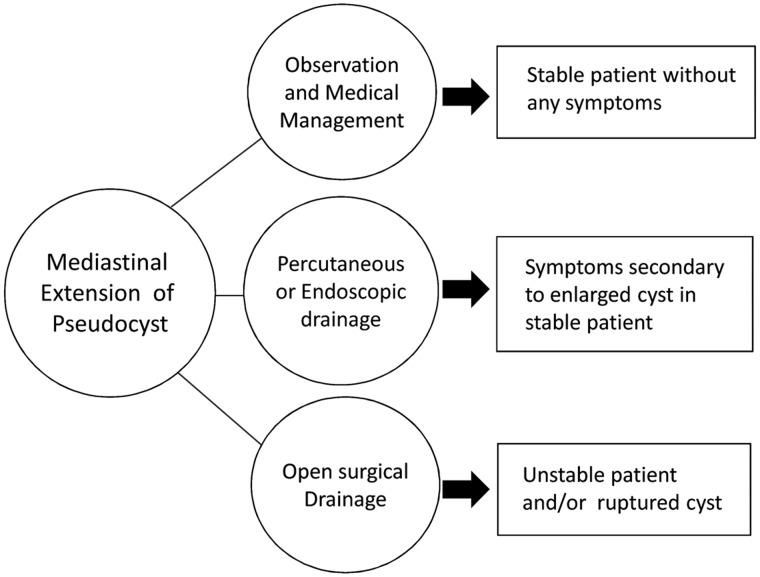
Management algorithm of MPP.

Management of MPP consists of drainage of the pancreatic fluid using various techniques (Figure 6). While endoscopic drainage using newer techniques such as EUS has shown some impressive results, only a few centers offer such procedures. Bhasin *et al.* have recently reported 12 cases of successful resolution of MPP with endoscopic transpapillary nasopancreatic drain (NPD) [26]. EUS and EUS-guided fine needle aspiration (FNA) also assist in identifying the extent of necrotic debris and thereby guiding the appropriate management. As mentioned earlier, endoscopic drainage is available only at a limited numbers of expert centers and further studies regarding the safety and efficacy of these techniques needs to be done. CT-guided percutaneous drainage is being used commonly, as it is readily available and gives the advantage of delineating the complex relationship of the cyst to the surrounding structures and thus minimizing complications. Catheter drainage is associated with better outcome and lower incidence of recurrence than needle aspiration [2]. Reported cases of mediastinal pseudocyst and the methods used to resolve the problem in the years 2008–2012 have been described in Table 2. [2, 9, 25–30].

**Table 2.** Reported cases of mediastinal pseudocyst and the methods used to resolve the problem in the years 2008–2012

No.	References	Age (years); gender	Symptoms	Solution
1	Panackel *et al.* 2008 [25]	35; male	Dysphagia	Octreotide and parenteral nutrition
2	Drescher *et al.* 2008 [9]	55; male	Dyspnea, dysphagia, weight loss	Laparotomy and external drainage through an abdominal access
3	Nuwayhid *et al.* 2011 [27]	4; male	Epigastric pain, vomiting, dysphagia	CT-guided percutaneous drainage via a posterior, extrapleural approach
4	Ajmera *et al.* 2012 [2]	43; male	Dysphagia, chest pressure	CT-guided drainage from left post side of thoracic spine
5	Gornals *et al.* 2012 [28]	37; male	Abdominal pain	EUS-guided drainage using a novel lumen-apposing metal stent
6	Kobayashi *et al.* 2012 [29]	62; male	Dyspnea	CT-guided puncture and endoscopic pancreatic drainage
7	Rana *et al.* 2012 [30]	42; male	Abdominal pain, shortness of breath, leuritic chest pain	Endoscopic transpapillary drainage
8	Bhasin *et al.* 2012 [26]	21–52; 10 male & 2 female	-	endoscopic transpapillary nasopancreatic drain

Surgical treatment is currently performed only rarely and should be reserved for patients with life-threatening presentations, ruptured cyst, failure of other less-invasive management, multiple strictures, complete pancreatic duct obstruction, and very large cysts [31]. Surgical options are internal drainage, external drainage, cyst-enterostomy or pancreatic resection [15].

Complications associated with percutaneous drainage include hemorrhage, infection and occasionally the formation of a fistula. Percutaneous drainage has a higher rate of recurrence and complications than EUS-guided drainage. Complications associated with EUS-guided drainage include hemorrhage, infection, esophageal stricture, post-procedure pancreatitis, stent migration and duodenal erosion [2, 30].

Formation of a pseudoaneurysm in the setting of acute and chronic pancreatitis has been described in the literature, with a reported rate of between 3.5–10% of patients with pancreatitis [32, 33]. Chronic and ongoing alcohol consumption is the most common etiological factor for the development of pseudoaneurysm [34]. Several mechanisms for their formation are described in the literature, including vessel wall erosion by a pseudocyst due to uncontrolled, severe inflammation necrotizing the vessel and release of the pancreatic enzymes, leading to proteolytic digestion of the walls of peri- and intra-pancreatic vessels [32, 35]. The most commonly involved blood vessel is the splenic artery (40%), followed by the gastroduodenal (30%), pancreaticoduodenal (20%), gastric (5%), and hepatic arteries (2%) [32]. Diagnosis is usually made with CT scan, revealing contrast enhancement within or adjacent to a suspected pseudocyst. Angiography remains the ‘gold standard' for exact localization of the site. The most severe complication of pseudoaneurysm is bleeding. Trans-catheter embolization is the preferred treatment option for hemodynamically stable patients who have evidence of bleeding on angiography. Immediate surgery is needed for unstable patients [32].

We report a unique case of MPP with left gastric artery pseudoaneurysm. In our case it was crucial to perform embolization of the gastric artery pseudoaneurysm prior to percutaneous drainage, in order to prevent hemorrhagic complications.

**Note:** This case was submitted as an abstract for poster presentation at the American College of Gastroenterology 2011 annual scientific meeting.

*Conflict of interest statement*: none declared.

## References

[1] Banks PA Bollen TL Dervenis C Classification of acute pancreatitis; 2012: revision of the Atlanta classification and definitions by international consensus. Gut 2013;62:102–11. 23100216 10.1136/gutjnl-2012-302779

[2] Ajmera AV Judge TA Mediastinal extension of pancreatic pseudocyst: a case with review of topic and management guidelines. Am J Ther 2012;19:e152–6. 21139451 10.1097/MJT.0b013e3181f62947

[3] Andrén-Sandberg A Dervenis C Pancreatic pseudocysts in the 21st century. Part I: classification, pathophysiology, anatomical considerations and treatment. JOP 2004;5:8–24. 14730118

[4] Gupta R Munoz JC Garg P Mediastinal pancreatic pseudocyst: a case report and review of the literature. MedGenMed 2007;9:8. 17955064 PMC1994836

[5] Johnston RH Owensby LC Vargas GM Pancreatic pseudocyst of the mediastinum. Ann Thorac Surg 1986;41:210–12. 3947175 10.1016/s0003-4975(10)62672-0

[6] Bhasin DK Rana SS Chandail VS Successful resolution of a mediastinal pseudocyst and pancreatic pleural effusion by endoscopic nasopancreatic drainage. JOP 2005;6:359–64. 16006688

[7] Komtong S Chanatrirattanapan R Kongkam P Mediastinal pseudocyst with pericardial effusion and dysphagia treated by endoscopic drainage. JOP 2006;7:405–10. 16832138

[8] Obuszko Z Beggs D Dysphagia due to pancreatic pseudocyst with mediastinal extension. Eur J Cardiothorac Surg 1998;13:316–8. 9628385 10.1016/s1010-7940(98)00004-9

[9] Drescher R Köster O Lukas C Mediastinal pancreatic pseudocyst with isolated thoracic symptoms: a case report. J Med Case Rep 2008;2:180. 18505589 10.1186/1752-1947-2-180 PMC2415357

[10] Colarian JH Sekkarie M Rao R Pancreatic pseudocyst mimicking idiopathic achalasia. Am J Gastroenterol 1998;93:103–5. 9448185 10.1111/j.1572-0241.1998.103_c.x

[11] Săftoiu A Ciurea T Dumitrescu D Endoscopic ultrasound-guided transesophageal drainage of a mediastinal pancreatic pseudocyst. Endoscopy 2006;38:538–9. 16767595 10.1055/s-2006-925229

[12] Tan MH Kirk G Archibold P Cardiac compromise due to a pancreatic mediastinal pseudocyst. Eur J Gastroenterol Hepatol 2002;14:1279–82. 12439127 10.1097/00042737-200211000-00020

[13] Lee FY Wang YT Poh SC Congestive heart failure due to a pancreatic pseudocyst. Cleve Clin J Med 1994;61:141–3. 8194180 10.3949/ccjm.61.2.141

[14] Bardia A Stoikes N Wilkinson NW Mediastinal pancreatic pseudocyst with acute airway obstruction. J Gastrointest Surg 2006;10:146–50. 16368505 10.1016/j.gassur.2005.05.009

[15] Zhang A Zheng S Treatment of pancreatic pseudocysts in line with D'Egidio's classification. World J Gastroenterol 2005;11:729–32. 15655832 10.3748/wjg.v11.i5.729 PMC4250749

[16] Leechawengwong M Berger HW Romeu J Spontaneous resolution of mediastinal pancreatic pseudocyst. Chest 1979;75:632–3. 436498 10.1378/chest.75.5.632

[17] Santoshkumar S Seith A Rastogi R Mediastinal pseudocysts in chronic pancreatitis with spontaneous resolution. Trop Gastroenterol 2007;28:32–4. 17896608

[18] Frenzer A Schubarth P Soucek M Disappearance of a large mediastinal pseudocyst in a patient with chronic alcoholic pancreatitis after total parenteral nutrition. Eur J Gastroenterol Hepatol 1995;7:369–71. 7600145

[19] Singh P Holubka J Patel S Acute mediastinal pancreatic fluid collection with pericardial and pleural effusion. Complete resolution after treatment with octreotide acetate. Dig Dis Sci 1996;41:1966–71. 8888708 10.1007/BF02093597

[20] Ishibashi K Suzuki T Isoo Y A case of mediastinal pancreatic pseudocysts successfully treated with somatostatin analogue. Nihon Shokakibyo Gakkai Zasshi 1999;96:176–80. 10087891

[21] Yasuda H Ino Y Igarashi H A case of pancreatic pleural effusion and mediastinal pancreatic pseudocyst: management by a somatostatin analogue octreotide. Pancreas 1999;19:410–2. 10547204 10.1097/00006676-199911000-00015

[22] Akashi T Kawabe K Sakamoto R A case of mediastinal pancreatic pseudocysts conservatively treated with somatostatin analogue. Nihon Shokakibyo Gakkai Zasshi 2003;100:713–8. 12833868

[23] Tsujimoto T Takano M Tsuruzono T Mediastinal pancreatic pseudocyst caused by obstruction of the pancreatic duct was eliminated by bromhexine hydrochloride. Intern Med 2004;43:1034–8. 15609697 10.2169/internalmedicine.43.1034

[24] Sakamoto Y Miura S A case of mediastinal pancreatic pseudocyst with hemoptysis successfully treated with total parenteral nutrition. Nihon Kokyuki Gakkai Zasshi 2007;45:582–6. 17682472

[25] Panackel C Korah AT Krishnadas D Pancreatic pseudocyst presenting as dysphagia: a case report. Saudi J Gastroenterol 2008;14:28–30. 19568491 10.4103/1319-3767.37801 PMC2702880

[26] Bhasin DK Rana SS Rao C Clinical presentation, radiological features, and endoscopic management of mediastinal pseudocysts: experience of a decade. Gastrointest Endosc 2012;76:1056–60. 22867447 10.1016/j.gie.2012.06.021

[27] Nuwayhid Z Kassira N Neville HL Percutaneous retropleural drainage of a posttraumatic pancreatic mediastinal pseudocyst in a child. J Pediatr Surg 2011;46:585–7. 21376216 10.1016/j.jpedsurg.2010.12.006

[28] Gornals JB Loras C Mast R Endoscopic ultrasound-guided transesophageal drainage of a mediastinal pancreatic pseudocyst using a novel lumen-apposing metal stent. Endoscopy 2012;44 Suppl 2:E211–2. 22622750 10.1055/s-0032-1309384

[29] Kobayashi S Uehara Y Oshima R A case of mediastinal pancreatic pseudocyst accompanied by pancreatic pleural effusion. Nihon Shokakibyo Gakkai Zasshi 2012;109:638–43. 22481266

[30] Rana SS Bhasin DK Rao C Esophageal stricture following successful resolution of a mediastinal pseudocyst by endoscopic transpapillary drainage. Endoscopy 2012;44 Suppl 2:E121–2. 22477176 10.1055/s-0031-1291695

[31] Sieber R Jermini-Gianinazzi I Del Grande F Mediastinal pancreatic pseudocyst and pleural effusion. Eur J Emerg Med 2011;18:180–1. 21540664 10.1097/MEJ.0b013e3283413094

[32] Mallick IH Winslet MC Vascular complications of pancreatitis. JOP 2004;5:328–37. 15365199

[33] White AF Baum S Buranasiri S Aneurysms secondary to pancreatitis. AJR Am J Roentgenol 1976;127:393–6. 183522 10.2214/ajr.127.3.393

[34] Udd M Leppäniemi AK Bidel S Treatment of bleeding pseudoaneurysms in patients with chronic pancreatitis. World J Surg 2007;31:504–10. 17322972 10.1007/s00268-006-0209-z

[35] Vanlangenhove P Defreyne L Kunnen M Spontaneous thrombosis of a pseudoaneurysm complicating pancreatitis. Abdom Imaging 1999;24:491–3. 10475934 10.1007/s002619900546

